# Foodborne Illness Acquired in the United States—Unspecified Agents

**DOI:** 10.3201/eid1701.P21101

**Published:** 2011-01

**Authors:** Elaine Scallan, Patricia M. Griffin, Frederick J. Angulo, Robert V. Tauxe, Robert M. Hoekstra

**Affiliations:** Author affiliation: Centers for Disease Control and Prevention, Atlanta, Georgia, USA

**Keywords:** Food poisoning, gastroenteritis, diarrhea, population surveillance, incidence estimates, United States, epidemiology, bacteria, viruses, research

## Abstract

Each year, 31 major known pathogens acquired in the United States caused an estimated 9.4 million episodes of foodborne illness. Additional episodes of illness were caused by unspecified agents, including known agents with insufficient data to estimate agent-specific illness, known agents not yet recognized as causing foodborne illness, substances known to be in food but of unproven pathogenicity, and unknown agents. To estimate these additional illnesses, we used data from surveys, hospital records, and death certificates to estimate illnesses, hospitalizations, and deaths from acute gastroenteritis and subtracted illnesses caused by known gastroenteritis pathogens. If the proportions acquired by domestic foodborne transmission were similar to those for known gastroenteritis pathogens, then an estimated 38.4 million (90% credible interval [CrI] 19.8–61.2 million) episodes of domestically acquired foodborne illness were caused by unspecified agents, resulting in 71,878 hospitalizations (90% CrI 9,924–157,340) and 1,686 deaths (90% CrI 369–3,338).

## Abstract 2

Each year, unspecified agents caused an estimated 38.4 million episodes of illness, resulting in 71,878 hospitalizations and 1,686 deaths.

Foodborne diseases are a major cause of illness and death in the United States. In another article, we estimated that each year, major known pathogens acquired in the United States caused 9.4 million episodes of foodborne illness, resulting in 55,961 hospitalizations and 1,351 deaths (*1*). (Hereafter, episodes of illness are referred to as illnesses.) Although the number of illnesses caused by these pathogens is substantial, these illnesses represent only a subset of the total illnesses.

An additional proportion of foodborne illness is probably caused by a heterogeneous group of less understood agents. First, many agents that cause acute gastroenteritis are recognized as known or possible causes of foodborne illness, but because of a paucity of data, the number of agent-specific illnesses cannot be estimated. This category includes infectious agents (e.g., *Aeromonas* spp., *Edwardsiella* spp., and *Plesiomonas* spp.) and noninfectious agents (e.g., mushroom and marine biotoxins, metals, and other inorganic toxins). Second, some known agents may not be recognized as being transmitted in food. Detection of *Clostridium difficile* in retail meat products suggests that it may sometimes be transmitted by that route (*2*), and foodborne transmission of *Trypanosoma* spp. has recently been recognized in Brazil but not in the United States (*3*). Third, microbes, chemicals, and other substances known to be in food could at some time be shown to cause acute illness. Fourth, agents of foodborne illness continue to be discovered. Many major foodborne pathogens, e.g., *Campylobacter* spp. and *Escherichia coli* O157, were recognized only in recent decades (*4**,**5*). For some outbreaks (e.g., Brainerd diarrhea), even when specimens are obtained quickly, no causative agent can be identified (*6**,**7*). Additional agents of foodborne illness probably remain undescribed (*8*). This article provides estimates of foodborne gastroenteritis illnesses, hospitalizations, and deaths in the United States, other than those caused by the 31 major known pathogens considered in our companion article (*1*).

## Methods

**Figure 1:**
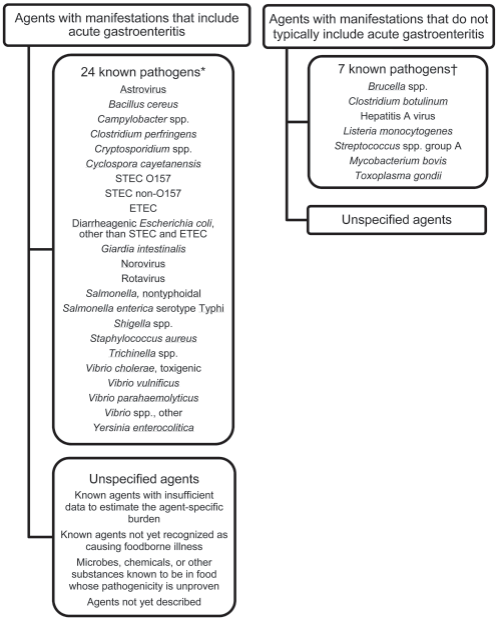
Agents that cause foodborne illness. STEC, Shiga toxin–producing *Escherichia coli*; ETEC, enterotoxigenic *E. coli*. *For most of these pathogens, the major manifestation is gastroenteritis. For some, i.e., *Salmonella enterica* serotype Typhi, *Trichinella* spp., and *Vibrio vulnificus*, some persons have diarrhea or vomiting, and the sign may initially look like those of gastroenteritis. †Most of these agents have major manifestations that do not typically include gastroenteritis. Diarrhea and vomiting can occur with some of these pathogens, e.g., *Clostridium botulinum* and hepatitis A virus, but are relatively uncommon. Only invasive *Listeria monocytogenes* infection, not diarrheal illness, is included in our estimates for known foodborne pathogens (*1*).

We defined unspecified agents as agents that cause acute gastroenteritis but that were not included in our estimate of foodborne illness caused by 31 major known pathogens (*1*). They include known agents with insufficient data for estimating agent-specific episodes of illness; known agents not yet recognized as causing foodborne illness; microbes, chemicals, or other substances known to be in food but for which pathogenicity is unproven; and agents not yet described. To estimate the extent of foodborne illness caused by unspecified agents, we estimated the number of acute gastroenteritis illnesses, hospitalizations, and deaths and subtracted the estimated number of acute gastroenteritis illnesses, hospitalizations, and deaths caused by 24 major known pathogens that typically or often cause diarrhea or vomiting (Figure 1). We refer to them as the 24 known gastroenteritis pathogens, although for a few, diarrhea or vomiting was not the main clinical sign. Estimates of illness were not made for unspecified agents that do not typically result in acute gastroenteritis.

We used data from the 24 known gastroenteritis pathogens to estimate the proportion of unspecified agents that were acquired in the United States (hereafter referred to as domestically acquired) and transmitted in food. Most of our data were from 2000 through 2007, and all estimates were based on the US population in 2006 (299 million persons) (*9*). To account for uncertainty, we used probability distributions to describe a range of plausible values for all model inputs. The modeling approach used and parameters of these probability distributions are detailed in the Technical Appendix. Our model outputs are in the form of probability distributions summarized by a mean point estimate with 90% credible intervals (CrIs).

### Acute Gastroenteritis

We estimated the number of episodes of acute gastroenteritis by using combined data from the Foodborne Diseases Active Surveillance Network (FoodNet) Population Surveys conducted in 2000–2001, 2002–2003, and 2006–2007 (Centers for Disease Control and Prevention [CDC], unpub. data). These methods are described in detail elsewhere (*10*). In brief, FoodNet Population Surveys are random-digit-dial telephone surveys of the general population in FoodNet sites. At the time of these surveys, the population in FoodNet sites included 11% (in 2000) to 15% (in 2007) of the US population. In 2005, the demographic features of this population were similar to those of the US population, but the proportion of Hispanics was lower (*11*).

Surveys were conducted over 12-month periods and collected information about episodes of diarrhea and vomiting in the past month. Our estimate of the annual number of episodes of acute gastroenteritis was derived by multiplying the average monthly prevalence by 12. An episode of acute gastroenteritis was defined as diarrhea (>3 loose stools in 24 hours) or vomiting in the past month, each lasting >1 day or resulting in restricted daily activities. We excluded persons with a chronic condition in which diarrhea or vomiting was a major clinical sign and persons with concurrent cough or sore throat. Data were weighted to compensate for unequal probabilities of selection and to reflect the surveillance population by age and sex.

The estimated rates of acute gastroenteritis according to individual surveys were 0.49 (2000–2001), 0.54 (2002–2003), and 0.73 (2006–2007) episodes per person per year. The number of episodes of acute gastroenteritis was estimated by applying the average rate (0.6 episodes/person/year) from the combined surveys to the 2006 US population estimate. Uncertainty was added by assuming that individual FoodNet site estimates were normally distributed with standard deviations equal to survey standard errors (Technical Appendix).

### Hospitalizations

We estimated the number of hospitalizations for acute gastroenteritis by using 2000–2006 national estimates from 3 sources: CDC National Center for Health Statistics (NCHS) National Hospital Discharge System (NHDS) (*12**,**13*); Healthcare Cost and Utilization Project, Nationwide Inpatient Sample (NIS) (*14*); and combined data from NCHS National Ambulatory and National Hospital Ambulatory Medical Care Surveys (NAMCS and NHAMCS) (*15*).

Codes from International Classification of Diseases, 9th Revision, Clinical Modification (ICD-9-CM), were used to extract hospital discharge data from NHDS and NIS when acute gastroenteritis was listed as 1 of the first 3 diagnoses. Acute gastroenteritis was defined as ICD-9-CM diagnostic codes 001–008 (infectious gastroenteritis of known cause), 009 (infectious gastroenteritis), 558.9 (other and unspecified noninfectious gastroenteritis and colitis), or 787.9 (other symptoms involving digestive system: diarrhea), excluding 008.45 (*C. difficile* colitis) and 005.1 (botulism). Many infectious diseases from which a pathogen was not isolated may be coded as other and unspecified noninfectious gastroenteritis and colitis. Annual national estimates from 2000–2006 NHDS and 2000–2006 NIS data were obtained by weighting the sample data according to NCHS and the Healthcare Cost and Utilization Project criteria (*12**,**14*).

To estimate hospitalizations for acute gastroenteritis from NAMCS and NHAMCS data, we combined data from both surveys and extracted patient visits to clinical settings (including physician offices, hospital emergency and outpatient departments) with a diagnosis of acute gastroenteritis resulting in hospitalization, when acute gastroenteritis was listed as 1 of the 3 codes. Acute gastroenteritis was defined by using the ICD-9-CM codes described above. Annual national estimates were obtained by weighting the sample data according to NCHS criteria (*15*).

During 2000–2006, mean annual rates of hospitalization for acute gastroenteritis were 203 hospitalizations per 100,000 persons according to NHDS data, 187 per 100,000 according to NIS data, and 109 per 100,000 according to NAMCS and NHAMCS data. To estimate the number of hospitalizations for acute gastroenteritis, we chose the PERT distribution with a low, modal, and high value determined by the lowest (90), mean (166), and highest (211) annual rate per 100,000 persons across all 3 surveys and applied this distribution to the 2006 US population (Technical Appendix).

### Deaths

We estimated the number of deaths caused by acute gastroenteritis by using multiple cause-of-death data from the National Vital Statistics System (2000–2006) (*16**,**17*) when acute gastroenteritis was listed as the underlying or a contributing cause. Acute gastroenteritis was defined as ICD, 10th Revision, codes A00.9–A08.5 (infectious gastroenteritis of known cause), A09 (diarrhea and gastroenteritis of presumed infectious origin), and K52.9 (noninfectious gastroenteritis and colitis, unspecified), excluding A04.7 (enterocolitis caused by *C. difficile*) and A05.1 (botulism). To estimate the number of deaths, we chose the mean death rate (1.5 deaths/100,000 population) as the modal value of a PERT distribution, used the lowest and highest annual death rates (1.2 and 2.4 deaths/100,000 population) as the lower and upper bounds, and applied this distribution to the 2006 US population.

### Domestically Acquired and Foodborne Acute Gastroenteritis

**Figure 2:**
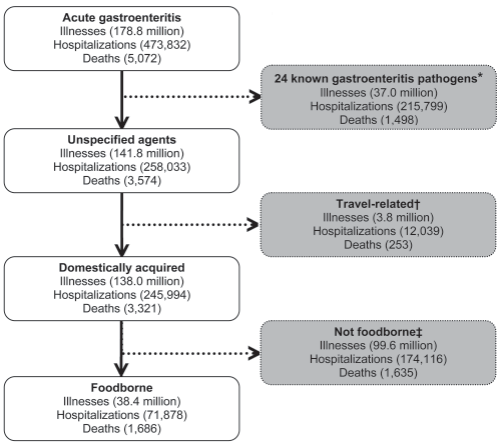
Schematic of estimates of illnesses, hospitalizations, and deaths caused by unspecified acute gastroenteritis agents. *The estimated numbers of illnesses, hospitalizations, and deaths (hereafter, illnesses refers to illnesses, hospitalizations, or deaths as appropriate) caused by the 24 known gastroenteritis pathogens (*1*) were subtracted to estimate the number of illnesses caused by unspecified agents. †The estimated numbers of illnesses related to travel were subtracted to estimate the number of domestically acquired illnesses. The estimates of the proportion related to travel were based on the overall weighted distribution of the proportions of illnesses that were related to travel from the 24 known gastroenteritis pathogens. ‡The estimated numbers of nonfoodborne illnesses were subtracted to estimate foodborne illnesses. The estimates of the proportion foodborne were based on the overall weighted distribution of the proportions of illnesses that were foodborne from the 24 known gastroenteritis pathogens. All estimates were based on US population in 2006.

To estimate acute gastroenteritis caused by unspecified agents, we subtracted the estimated number of illnesses, hospitalizations, and deaths caused by the 24 known gastroenteritis pathogens from our estimate of the overall number of illnesses, hospitalizations, and deaths from acute gastroenteritis (Figure 2). To estimate the number that were domestically acquired and transmitted by food, we used the overall weighted distribution of the proportions of illnesses, hospitalizations, and deaths that were domestically acquired and foodborne from the 24 known gastroenteritis pathogens to describe the lower, modal, and upper values of the PERT distribution and applied these separately to the estimates of unspecified illnesses, hospitalizations, and deaths (Technical Appendix).

### Uncertainty Analysis

The parametric distribution used for almost all multipliers was a 4-parameter beta (modified PERT) distribution (*18*). The first 3 parameters are low, modal, and high. The fourth parameter is related to the variability of the distribution. We typically fixed this last parameter at 4, which yields the simple PERT distribution (*18*). However, when describing the proportions domestically acquired and foodborne from the 24 known gastroenteritis pathogens, we used a value of 2 to reflect greater uncertainty (Technical Appendix). On the basis of 100,000 iterations, we obtained empirical distributions of counts corresponding to Bayesian posterior distributions and used these posterior distributions to generate a point estimate (posterior mean) and upper and lower 5% limits for 90% CrIs. We used SAS version 9.2 (SAS Institute, Cary, NC, USA) for these analyses.

## Results

### Foodborne Illnesses

We estimate that 38.4 million (90% CrI 19.8–61.3 million) episodes of domestically acquired foodborne gastroenteritis were caused by unspecified agents (Figure 2) as follows. We estimated that 178.8 million acute gastroenteritis illnesses occurred each year in the United States. Subtracting 37.0 million estimated illnesses caused by the 24 known gastroenteritis pathogens leaves 141.8 million acute gastroenteritis illnesses caused by unspecified agents. The proportion of these unspecified agents acquired through domestic foodborne transmission is unknown; however, applying the distribution of the proportion of illnesses from the 24 known gastroenteritis pathogens that were domestically acquired (98%) and foodborne (25%) yields an estimate of 38.4 million domestically acquired foodborne illnesses caused by unspecified agents.

### Hospitalizations

We estimate that 473,832 hospitalizations resulted from acute gastroenteritis each year in the United States (Figure 2). Subtracting the 215,799 estimated hospitalizations caused by the 24 known gastroenteritis pathogens leaves 258,033 hospitalizations for acute gastroenteritis caused by unspecified agents. The proportion of these unspecified agents that were acquired as a result of domestic foodborne transmission is unknown; however, applying the distributions of the proportion of hospitalizations among the 24 known gastroenteritis pathogens that were domestically acquired (97%) and foodborne (23%) yields an estimate of 71,878 hospitalizations (90% CrI 9,924–157,340) caused by domestically acquired unspecified agents that were transmitted by food.

### Deaths

We estimate that an estimated 5,072 persons died of acute gastroenteritis each year in the United States (Figure 2). Subtracting the 1,498 deaths caused by the 24 known gastroenteritis pathogens leaves 3,574 acute gastroenteritis deaths caused by unspecified agents. The proportion of these unspecified agents acquired as a result of domestic foodborne transmission is unknown; however, applying the distributions of the proportion of deaths among the 24 known gastroenteritis pathogens that were domestically acquired (95%) and foodborne (50%) yields an estimate of 1,686 (90% CrI 369–3,338) deaths caused by domestically acquired unspecified agents that were transmitted by food.

## Discussion

Unspecified agents are major contributors to the total number of episodes of acute gastroenteritis and foodborne diseases. If distribution of domestically acquired and foodborne agents is similar to that of the 24 known gastroenteritis pathogens (*1*), then these agents cause 38.4 million episodes of foodborne gastroenteritis each year in the United States, resulting in 78,878 hospitalizations, and 1,686 deaths. Combining the estimates for unspecified agents and major known pathogens provides an estimate of the total effect of contaminated food consumed in the United States: 47.8 million episodes of illness, 127,839 hospitalizations, and 3,037 deaths (Table).

**Table:** Estimated annual number of episodes of domestically acquired, foodborne illness, hospitalizations, and deaths caused by 31 pathogens and unspecified agents transmitted through food, United States*

Cause	Illnesses			Hospitalizations			Deaths	
	Mean (90% CrI)	%		Mean (90% CrI)	%		Mean (90% CrI)	%
Major known pathogens†	9,388,075 (6,641,440–12,745,709)	20		55,961 (39,534–75,741)	44		1,351 (712–2,268)	44
Unspecified agents‡	38,392,704 (19,829,069–61,196,274)	80		71,878 (9,924–157,340)	56		1,686 (369–3,338)	56
Total	47,780,779 (28,658,973–71,133,833)	100		127,839 (62,529–215,562)	100		3,037 (1,492–4,983)	100

*All estimates were based on US population in 2006. CrI, credible interval. †The 31 known pathogens are astrovirus, *Bacillus cereus*, *Brucella* spp., *Campylobacter* spp., *Clostridium botulinum*, *Clostridium perfringens*, *Cryptosporidium* spp., *Cyclospora cayetanensis*, enterotoxigenic *Escherichia coli* (ETEC), Shiga toxin–producing *E. coli* (STEC) O157, STEC non-O157, diarrheagenic *E. coli* other than STEC and ETEC, *Giardia intestinalis*, hepatitis A virus, *Listeria monocytogenes, Mycobacterium bovis*, norovirus, rotavirus, sapovirus, nontyphoidal *Salmonella* spp., *S. enterica* serotype Typhi, *Shigella* spp., *Staphylococcus aureus, Streptococcus* spp. group A, *Toxoplasma gondii*, *Trichinella spp.*, *Vibrio cholerae*, *V. vulnificus*, *V. parahemolyticus*, other *Vibrio* spp., and *Yersinia* spp (*1*). ‡Unspecified agents are defined as agents that cause acute gastroenteritis other than the 31 major known pathogens listed above. They include known agents with insufficient data to estimate agent-specific episodes of illness; known agents not yet recognized as causing foodborne illness; microbes, chemicals, and other substances known to be in food but whose pathogenicity is unproven; and agents not yet described.

Our estimate of foodborne illness caused by unspecified agents is lower than that estimated by CDC in 1999 (38.4 million vs. 62 million, respectively) (*19*). A major reason for this decrease is our lower estimate of episodes of acute gastroenteritis, which probably resulted from changes in data sources and methods rather than a real decline in the rate of illness. Our estimate is derived from the 3 most recent FoodNet Population Surveys, which had a sample size 5× greater than that in the 1996–1997 FoodNet survey used for the 1999 estimates. Additionally, the 1999 estimates relied on respiratory symptom and vomiting data from US studies conducted before 1980 (*20**,**21*). The current and the 1999 estimates excluded persons reporting concurrent cough or sore throat, but the proportion of respondents reporting these signs was higher in the current than in the earlier surveys (38% vs. 25%), contributing to a lower estimated prevalence of acute gastroenteritis (0.60 vs. 0.79 episodes/person/year). In addition, the current study excluded persons with vomiting who had been ill for <1 day or whose illness did not result in restricted daily activities, whereas the 1999 estimate included all persons with vomiting. All these factors contributed to the current estimate of acute gastroenteritis being 24% lower than the 1999 estimate.

The proportion of illnesses estimated to be foodborne was also a major driver of the current lower estimate of illness caused by unspecified foodborne agents. Because no data existed with which to directly estimate the proportions of unspecified agents that were domestically acquired and foodborne, distributions of these proportions were estimated to be similar to those of the 24 known gastroenteritis pathogens (*1*). Because norovirus accounts for 59% of illnesses caused by the 24 known gastroenteritis pathogens, the foodborne proportion was driven largely by norovirus. The proportion of foodborne norovirus used for the current estimate is 26%, a marked decrease from 40% used for the 1999 estimates. Additionally, unlike the 1999 estimates, the current estimates exclude international travel-related illnesses. As a result of these newer data and revised methods, the mean proportion of unspecified agents that were estimated to be transmitted by food was 25%, which is lower than 36% used for the 1999 estimate.

Estimating the number of hospitalizations and deaths caused by unspecified foodborne agents is challenging. For an illness caused by a pathogen to be recorded, a physician must order the appropriate diagnostic tests and the pathogen must be detected. Without identification of a pathogen, infections producing signs and symptoms of gastroenteritis may be coded as nonspecific signs or symptoms or as noninfectious illnesses (*22*). Our overall estimates of hospitalizations and deaths from acute gastroenteritis, derived from national data sources, include codes for infectious and noninfectious gastroenteritis. To avoid overestimating the number of hospitalizations, we selected outpatient visits resulting in hospitalization and we selected hospital discharge records on the basis of only the first 3 listed diagnoses. This approach was a compromise between limiting the analysis to hospitalizations for which acute gastroenteritis was listed as the primary cause and including hospitalizations for which signs or symptoms of gastroenteritis may have been a manifestation of another illness. This approach has been taken in other studies (*23**,**24*). For deaths, we included all records in which acute gastroenteritis was listed as an underlying or a contributing cause.

Our approach to estimating illness caused by unspecified agents has many limitations. First, the accuracy of our estimate of the number of acute episodes of gastroenteritis from the FoodNet Population Surveys has not been validated. This estimate was based on responses to questions about diarrhea and vomiting in the past month. These data provide a measure of prevalent cases; however, we lack sufficient data on duration of illness, onset date, and multiple episodes in the past month necessary to estimate incidence. An analysis of the FoodNet Population Surveys reported a 2-day median duration of acute gastroenteritis (*10*), suggesting that the increase in the estimate of illnesses based on incidence versus prevalence would probably be small and would be included within the range of sampling variability and uncertainty associated with our estimate of acute gastroenteritis. The accuracy of the 1-month recall period for acute gastroenteritis is also unknown. Some evidence suggests that shorter recall periods (e.g., past week) may result in higher reported prevalence (*25*). Which recall period is more accurate is not known. Our estimate of the number of episodes of acute gastroenteritis may be too high. Although our survey attempted to eliminate other causes of illness by asking about chronic diseases, some of the vomiting illnesses classified as acute gastroenteritis, for example, could have been caused by medications, alcohol withdrawal, or other causes, and some of the diarrheal illnesses could be caused by medications or other causes. However, our criteria for acute gastroenteritis were fairly strict, and foodborne illnesses probably occurred in some persons who were excluded because they had concurrent cough or sore throat or because their illness lasted for only 1 day. Second, we may have underestimated the episodes of illness caused by the 24 known gastroenteritis pathogens. When our method is used, any increase in the estimate for the major known pathogens will result in a decrease in the estimate for unspecified agents. Recent serologic data from European countries suggest that infection with *Salmonella* spp. is more common than estimated by other methods, including ours; however, many of these infections may be asymptomatic (*26*). Finally, the proportion of illnesses transmitted by food for unspecified agents is unknown and may differ from that for the 24 known gastroenteritis pathogens. Studies estimating foodborne disease in England and Wales and in Australia (which also attributed a large proportion of foodborne illness to unspecified agents: 73% in Australia and 48% in England and Wales vs. 80% in the United States) have estimated a similar proportion of acute gastroenteritis episodes to be transmitted by food (32%, and 26%, respectively vs. 25% in the United States) (*27**,**28*). A study of illness caused by known chemical agents, with estimates of the proportion that include acute gastroenteritis and that are foodborne, could help improve these estimates.

Combining the estimate for unspecified agents with that for the 31 major known pathogens to arrive at an estimate of overall foodborne illness has limitations. The method used for unspecified agents began with an estimate of acute gastroenteritis episodes, hospitalizations, and deaths and scaled down to a number for domestically acquired foodborne illnesses, hospitalizations, and deaths. Conversely, for most known pathogens, our estimate scaled counts of laboratory-confirmed illnesses up to an estimated number of ill persons, accounting for underreporting and underdiagnosis factors that contribute to an illness not being reported to public health agencies. Combining different approaches is not optimal because the methods themselves may affect the estimates derived. Also, our estimates do not include unspecified foodborne illnesses that do not typically cause signs of acute gastroenteritis. Most foodborne outbreak–associated illnesses caused by chemical agents reported to CDC during 2001–2006 (*29*) were not due to agents characterized by acute gastroenteritis and so would not be included in our estimates.

Although the number of episodes of foodborne disease caused by unspecified agents is substantial, the claim that 80% of foodborne illnesses are unspecified must be treated with caution. Illnesses caused by the 24 known gastroenteritis pathogens were, in most instances, estimated by using models that scaled counts of laboratory-confirmed illnesses up to an estimated number of illnesses with aggregate multipliers to adjust for underreporting and underdiagnosis factors that contribute to an illness not being reported to public health agencies (*1*). These multipliers are sensitive to the methods and modeling approaches used, and different choices could have increased estimates for the 24 known gastroenteritis pathogens, thus decreasing the estimate of foodborne illness caused by unspecified agents. For example, we took a conservative approach to estimating the underreporting multiplier for pathogens for which illness counts were derived from outbreak data (*1*); a less conservative approach would have increased estimated illnesses for these pathogens.

Future estimates might be improved by validating them by using other data on acute gastroenteritis episodes, hospitalizations, and deaths, such as by reviewing acute gastroenteritis coded as a secondary discharge diagnosis or assessing the accuracy of acute gastroenteritis coding on death certificates. The diagnostic gap might be narrowed by identifying additional agents linked to foodborne transmission. Systematic laboratory investigation of specimens from well-investigated outbreaks of foodborne disease of undetermined cause, and detailed investigations of specific syndromes, may identify new agents (*4**,**5**,**30*).

## Supplementary Material

Appendix Model Structure for Estimating Foodborne Illness Caused by Unspecified Agents.
