# Supplementary material for: Foodborne Illness Acquired in the United States—Unspecified Agents
Source: Emerg Infect Dis. 2011 Jan;17(1):16–22. doi: 10.3201/eid1701.P21101 (PMC3204615; doi:10.3201/eid1701.P21101)
Supplement: Appendix — Model Structure for Estimating Foodborne Illness Caused by Unspecified Agents. [file 09-1101p2-Techapp.pdf]

Article DOI: 10.3201/eid1701.P21101

Foodborne Illness Acquired in the United States—Unspecified Agents

Technical Appendix

Model Structure for Estimating Foodborne Illness Caused by Unspecified Agents

Background

As described in the introduction, this paper estimates the burden of acute gastroenteritis (AGI) illnesses, hospitalizations, and deaths caused by agents other than the 31 major known pathogens considered in Scallan et al. in the first part of this two-part research report in this issue of Emerging Infectious Diseases (*1*). The model for the outcomes in this second report builds directly on the models created to estimate the burden of the 31 major known pathogens, details of which are available in part 1 of this paper and in online Technical Appendix 2 (www.cdc.gov/EID/content/17/1/7-Techapp2.pdf) (*1*). Our description below assumes familiarity with that material.

Model Structures

The problem of determining the burden due to unspecified agents was approached in a stepwise manner.

The real first step required identification of known agents that cause AGI. For 21 of the 31 major known pathogens of foodborne illness, AGI was considered a major manifestation (e.g., *Campylobacter, Salmonella* nontyphoidal). An additional 3 pathogens (i.e., *Salmonella* serotype Typhi, *Trichinella* spp., and *Vibrio vulnificus*) often cause diarrhea or vomiting, and the disease can initially manifest as AGI; therefore, these were included among the "gastroenteritis" pathogens, for a total of 24 known gastroenteritis pathogens. Seven pathogens were considered to have major manifestations that do not typically include AGI. Although diarrhea and vomiting can occur with some of these (e.g., *Clostridium botulinum* and hepatitis A virus), the gastroenteritis manifestations were considered relatively uncommon.

Having identified 24 known gastroenteritis pathogens, it is useful to summarize the problem and the data available to solve it.

The problem can be described with the following relationship:

Total AGI = AGI caused by known pathogens + AGI caused by unspecified agents

We use the term AGI to refer generically to acute gastroenteritis illnesses, illnesses that result in hospitalization, and illnesses that result in death, when appropriate.

Knowledge of any 2 terms yields the third. We have some information about total AGI and about AGI caused by known pathogens. Specifically, we have survey data to estimate rates of AGI illnesses, hospitalizations, and deaths. And we have 24 sets of 100,000 Monte Carlo simulated observations for outcomes corresponding to the 24 known gastroenteritis pathogens. Each observation contains values for each of the following 9 outcomes: numbers of illnesses, hospitalizations, and deaths; domestically acquired illnesses, hospitalizations, and deaths; and domestically acquired foodborne illnesses, hospitalizations, and deaths.

The unspecified agents model can be described as consisting of the following 5 steps:

1. **Simulate total AGI**: Simulate 100,000 observations to obtain distributions for total acute gastroenteritis illnesses, hospitalizations, and deaths based on rates estimated from population surveys.
2. **Sum the known counts**: Sum the counts from all 9 outcomes across the 24 known gastroenteritis pathogens. This will yield 100,000 observations representing the distributions of the 9 outcomes for the sum of the known pathogens. For each of those 100,000 observations, using ratios of outcome values, compute the separate percentages of illnesses, hospitalizations, and deaths that were domestically acquired, and compute the separate percents of illnesses, hospitalizations, and deaths that were foodborne among those that were domestically acquired.
3. **Fit PERTs to known sum**: Fit PERT distributions using maximum likelihood to the simulated data for each of the 6 percentages that were computed in step 2.
4. **Adjust the PERTs and apply to AGI**: Take the min, modal, max, and variance parameters from step 3. Change the variance parameters to 2. Use the resulting

PERT distributions for the percentages and Monte Carlo simulation to generate counts of domestically acquired total AGI and domestically acquired foodborne AGI for each observation from step 1.

5. **Subtract**: Subtract the counts for each of the 9 outcomes for the 24 known gastroenteritis pathogens from the corresponding numbers for total AGI.

Figure 1 illustrates the steps schematically. Note that the steps required some different approaches from those used for the 31 major known pathogens considered in the first part of this research report (*1*). Specific model inputs and parameterizations are described in the Table (next page).

Figure 1: Schematic illustration for the method used to estimate AGI due to unspecified acute gastroenteritis agents

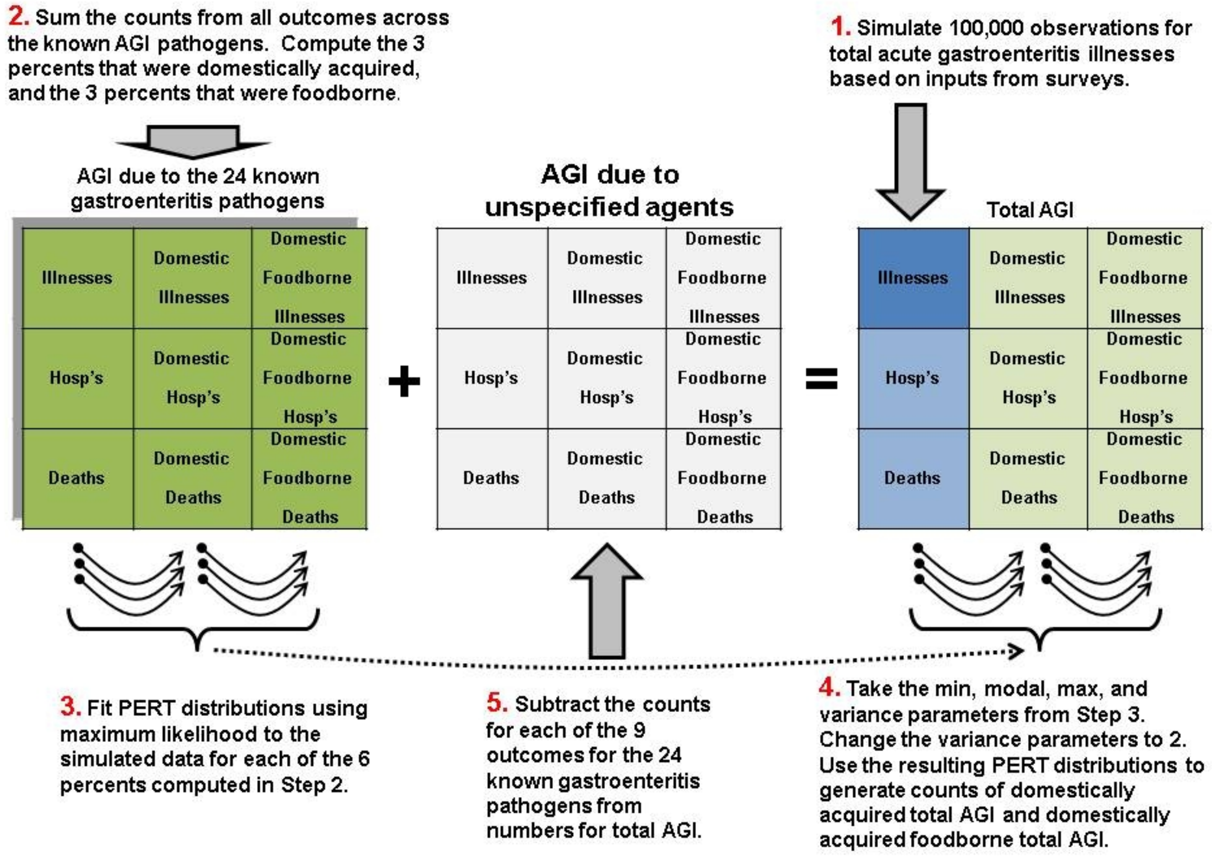

Table. Model inputs for estimating illnesses, hospitalizations, and deaths due to unspecified acute gastroenteritis agents			
**Model input**	**Data source(s)**	**Distribution**	**Parameters**
Population at risk	Estimated using 2006 US Census population estimate.	Constant	299,000,000
Acute gastroenteritis illnesses	Estimated rate per person per year by site using combined data from FoodNet Population Surveys in 2000–2001 (0.49 per person per year), 2002–2003 (0.54 per person per year), and 2006–2007 (0.73 per person per year) (CDC, unpublished data). Uncertainty from the site-specific survey estimates was added by assuming that site estimates were normally distributed with standard deviations equal to survey standard errors.	Mixture of normals	By FoodNet site: 0.61, 0.63, 0.51, 0.68, 0.51, 0.56, 0.63, 0.63, 0.56, 0.65
Proportion hospitalized	Estimated rate per 100,000 using annual national estimates from the 2000–2006 National Hospital Discharge System (mean: 203 per 100,000) (*3*), the 2000-2006 Nationwide Inpatient Sample (mean: 187 per 100,000) (*4*), and data from the 2000-2006 National Ambulatory Medical Care and National Hospital Ambulatory Medical Care Surveys (mean: 208 per 100,000) (*5*). Low, modal, and high values were determined using the lowest (148), mean (199), and highest (257) annual rate per 100,000.	PERT	Low, modal, high values: 0.0025, 0.0033, 0.0043
Proportion who died	Estimated annual rate per 100,000 persons using data from 2000–2006 multiple cause-of-death data from the Nation Vital Statistics System (*6*). Low, modal, and high values were determined using the lowest (1.2), mean (1.5), and highest (2.4) annual rate per 100,000.	PERT	Low, modal, high values: 0.00002, 0.000026, 0.00004
Posterior distributions of aggregate outcomes from the 24 known gastroenteritis pathogens	Individual pathogen posterior distributions from part 1 of this research report (*1*).	Monte Carlo simulation	Nonparametric
Percentage domestically acquired among overall acute gastroenteritis illnesses	Ratios of domestically acquired to total illnesses from aggregate distribution of the 24 known gastroenteritis pathogens. PERT parameters estimated by maximum likelihood and variance parameter subsequently set equal to 2. The initial variance parameter estimate is also indicated under Parameters.	PERT	Low, modal, high, and [variance] values: 0.950, 0.979, 0.993, [12 => 2]
Percentage domestically acquired among overall acute gastroenteritis hospitalizations	Ratios of domestically acquired to total hospitalizations from aggregate distribution of the 24 known gastroenteritis pathogens. PERT parameters estimated by maximum likelihood and variance parameter subsequently set equal to 2. The initial variance parameter estimate is also indicated under Parameters.	PERT	Low, modal, high, and [variance] values: 0.924, 0.969, 0.989, [11 => 2[
Percentage domestically acquired among overall acute gastroenteritis deaths	Ratios of domestically acquired to total deaths from aggregate distribution of the 24 known gastroenteritis pathogens. PERT parameters estimated by maximum likelihood and variance parameter subsequently set equal to 2. The initial variance parameter estimate is also indicated under Parameters.	PERT	Low, modal, high, and [variance] values: 0.826, 0.953, 0.997, [7 => 2]
Percentage foodborne among overall acute gastroenteritis illnesses	Ratios of foodborne to total illnesses from aggregate distribution of the 24 known gastroenteritis pathogens. PERT parameters estimated by maximum likelihood and variance parameter subsequently set equal to 2. The initial variance parameter estimate is also indicated under Parameters.	PERT	Low, modal, high, and [variance] values: 0.173, 0.251, 0.455, [19 => 2]
Percentage foodborne among overall acute gastroenteritis hospitalizations	Ratios of foodborne to total hospitalizations from aggregate distribution of the 24 known gastroenteritis pathogens. PERT parameters estimated by maximum likelihood and variance parameter subsequently set equal to 2. The initial variance parameter estimate is also indicated under Parameters.	PERT	Low, modal, high, and [variance] values: 0.139, 0.231, 0.474, [14 => 2]
Percentage foodborne among overall acute gastroenteritis deaths	Ratios of foodborne to total deaths from aggregate distribution of the 24 known gastroenteritis pathogens. PERT parameters estimated by maximum likelihood and variance parameter subsequently set equal to 2. The initial variance parameter estimate is also indicated under Parameters.	PERT	Low, modal, high, and [variance] values: 0.195, 0.495, 0.861, [5 => 2]

Selected Details and Considerations

Step 1: Simulate total AGI.

Step 1 is detailed in the main text and in online Technical Appendix 3 (www.cdc.gov/EID/content/17/1/7-Techapp2.pdf) under norovirus of Scallan et al. (*1*).

Step 2: Sum the known counts

- One issue that could come up is that of missing values. When summing 24 observations, an incomplete record in any dataset produces an incomplete record in the aggregate data. We resolved missing data issues by choosing data sources and series with few missing values, using simple imputation to fill in remaining missing values (*1*).
- The models for individual pathogens described in Scallan et al. (*1*) assumed that, for each pathogen, the percentages of illness, hospitalization, and death that were domestically acquired were equal. Similarly those models assumed that, for each pathogen, the percents of illness, hospitalization, and death that were foodborne were equal. Because the counts of the outcomes for the 24 pathogens vary, their sum reflects variable percentages for illness, hospitalization, and death. As a result we must calculate 6 percentages to simulate the complete set of outcomes for total AGI.
- No undefined ratios were encountered in computing percentages because the accumulated counts were large.

Step 3: Fit PERTs to known sum

- Step 3 is straightforward but can be computationally delicate because maximum likelihood fits of PERT/4-parameter beta distributions often have convergence problems, which result in no values for the parameters sought. The initial distributions were fit to 4-parameter beta distributions. The estimated parameters were then transformed to min, modal, max, and variance parameter values for the (modified) PERT distribution. Recall from online Technical Appendix 2 of the first part of this research report (*1*) that the distribution families are equivalent, with the PERT formulation, with its physically interpretable parameters, being

better for our usage. We were generally able to obtain convergence. When convergence was a problem in SAS 9.2, we used the minimum and maximum observed values of the data as fixed input parameters and solved for the remaining two parameters via maximum likelihood. We verified the robustness of these solutions using a second computing package (JMP 8.0.2, 2009 SAS Institute Inc), which implements a different algorithm, and yielded convergence for all 6 distributions. Other solutions to this problem could be tried; we did not think more sophisticated Bayesian methods were justified because of the degree of nonstatistical uncertainty in the inputs.

- Figure 2 (next page) shows the source data as histograms, and illustrates the initial 4-parameter beta/PERT fits as well as the modified fits that were applied to increase uncertainty, as described in step 4 below.

Figure 2. Estimation of 4-parameter Beta distributions and adjustment of corresponding PERT variance parameters to increase uncertainty

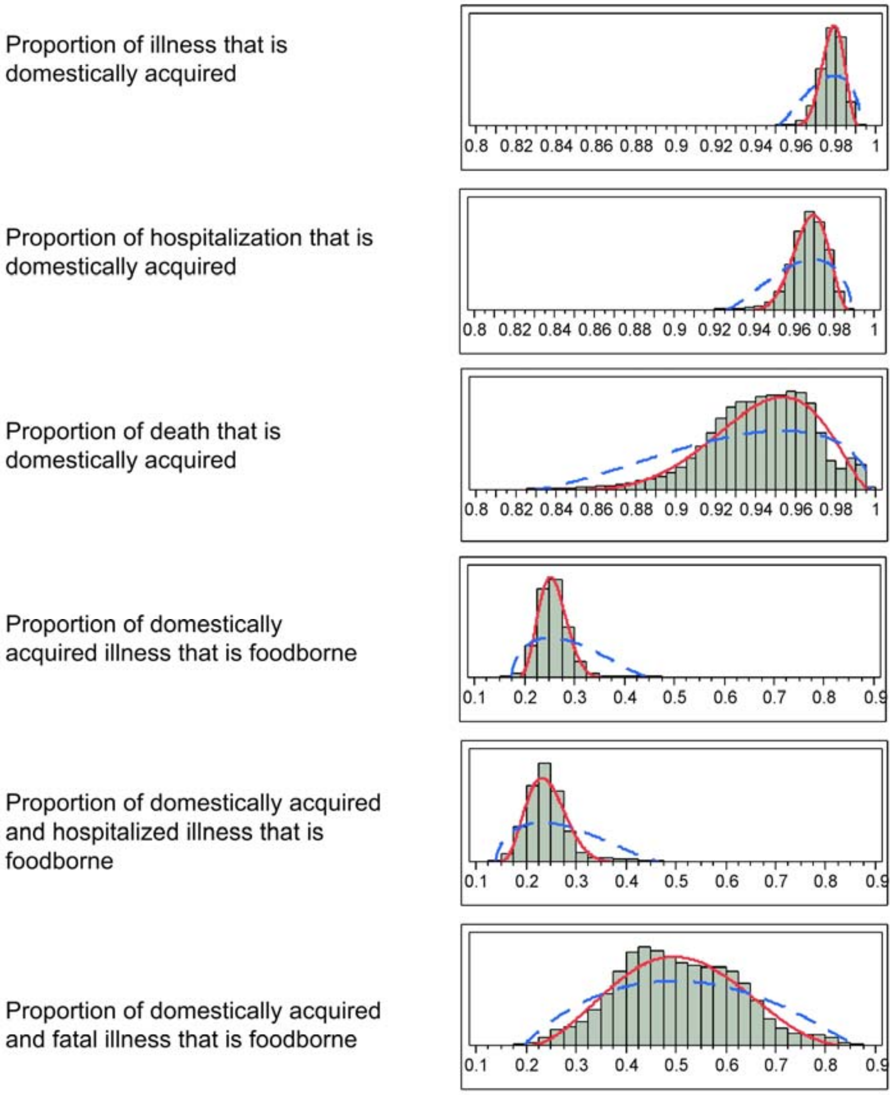

- The histograms represent the 100,000 simulated observations derived from summing outcomes for the 24 known gastroenteritis pathogens
- The red curves represent maximum likelihood fitted 4-parameter Beta distributions
- The dashed blue curves represent PERT distributions with low, modal, and high values that match the 4-parameter Beta distributions, but with variance parameters set equal to 2

Step 4: Adjust the PERTs and apply to AGI

- Step 4 involves setting the variance parameters of the 6 PERT distributions equal to 2. This increased the variance of all the distributions. The change was largest for illnesses and smallest for deaths (Table). We specifically chose not to change maximum and minimum values of the 4 PERT distributions, preferring to keep

the introduction of additional uncertainty as simple as possible and to avoid additional unneeded subjective inputs. For the same reasons we did not change the uncertainties of the 24 component distributions to achieve a more "uncertain" sum.

Step 5: Subtract

- Step 5 appears extremely simple but is not. At this stage we have 100,000 simulated observations for 9 outcomes for total acute gastroenteritis illnesses and 100,000 independently simulated observations for 9 outcomes for the 24 known gastroenteritis pathogens. Simple value-by-value subtraction is not valid because a number of negative values result. This happens because each of the 2 multivariate distributions is highly variable and the two 100,000 observation series are independent; this means that small values for a total AGI outcome occasionally line up with large values of a known pathogens outcome, with subtraction resulting in a negative value. This happened for about 4% of the simulated observations and only involved outcomes for hospitalization and death. We resolved this problem by adding a step to make selection of the values contingent. For any given observation for total acute AGI, an observation for known pathogens was selected at random from among those for which outcome values of the difference were positive. Given the small percentage of negative values initially generated, and their relatively small magnitudes, we chose not to use a more sophisticated approach. The problem of negative values could become more difficult when acute gastroenteritis illnesses due to known pathogens comprise a higher percentage of overall AGI. Then a more sophisticated approach, such as the use of copulas (*2*), would be necessary.

Final Comments

The methods discussion of Scallan et al. (*1*), detailed primarily in its online Technical Appendixes, emphasizes the need to discuss and incorporate both statistical and nonstatistical uncertainty. Because estimating foodborne illness caused by unspecified agents is a much more compact problem, we have presented a rather algorithmic discussion. The largest and most

tenuous assumption in the estimates for the unspecified agents is the assumption that features of the modeled disease process for the remaining acute gastroenteritis agents are identical to those for the aggregate 24 known gastroenteritis pathogens. Although we have used a fairly straightforward method, the specific choices are subjective, and we introduced nonstatistical uncertainty to reflect this.

References

1. Scallan E, Hoekstra RM, Angulo FJ, Tauxe RV, Widdowson M-A, Roy SL, et al. Foodborne illness acquired in the United States—major pathogens. Emerg Infect Dis. 2011;17:7–15.

2. Nelsen RB. An introduction to copulas. New York: Springer; 1999.

3. National Center for Health Statistics. National Hospital Discharge Survey [cited 2010 Feb 2].: http://www.cdc.gov/nchs/nhds.htm

4. Healthcare Cost and Utilization Project. National Inpatient Sample [cited 2010 Feb 8]. http://www.hcup-us.ahrq.gov/nisoverview.jsp

5. National Center for Health Statistics. Ambulatory health care data [cited 2010 Feb 8]. http://www.cdc.gov/nchs/ahcd/about_ahcd.htm

6. National Center for Health Statistics. Multiple cause-of-death public-use data files [cited 2010 Feb 8]. http://www.cdc.gov/nchs/products/elec_prods/subject/mortmcd.htm
